# Carbohydrate-Based Host-Guest Complexation of Hydrophobic Antibiotics for the Enhancement of Antibacterial Activity

**DOI:** 10.3390/molecules22081311

**Published:** 2017-08-08

**Authors:** Daham Jeong, Sang-Woo Joo, Vijay Vilas Shinde, Eunae Cho, Seunho Jung

**Affiliations:** 1 Department of Bioscience and Biotechnology, Microbial Carbohydrate Resource Bank (MCRB), Konkuk University, 120 Neungdong-ro, Gwangjin-gu, Seoul 05029, Korea; amir@konkuk.ac.kr (D.J.); sanchi900@gmail.com (S.-W.J.); vijay.shinde040@gmail.com (V.V.S.); 82goodgirl@hanmail.net (E.C.); 2 Center for Biotechnology Research in UBITA (CBRU), Institute for Ubiquitous Information Technology and Applications (UBITA), Konkuk University, 120 Neungdong-ro, Gwangjin-gu, Seoul 05029, Korea

**Keywords:** host-guest complex, antibiotics, cyclodextrin, cyclosophoraose, linear oligosaccharide

## Abstract

Host-guest complexation with various hydrophobic drugs has been used to enhance the solubility, permeability, and stability of guest drugs. Physical changes in hydrophobic drugs by complexation have been related to corresponding increases in the bioavailability of these drugs. Carbohydrates, including various derivatives of cyclodextrins, cyclosophoraoses, and some linear oligosaccharides, are generally used as host complexation agents in drug delivery systems. Many antibiotics with low bioavailability have some limitations to their clinical use due to their intrinsically poor aqueous solubility. Bioavailability enhancement is therefore an important step to achieve the desired concentration of antibiotics in the treatment of bacterial infections. Antibiotics encapsulated in a complexation-based drug delivery system will display improved antibacterial activity making it possible to reduce dosages and overcome the serious global problem of antibiotic resistance. Here, we review the present research trends in carbohydrate-based host-guest complexation of various hydrophobic antibiotics as an efficient delivery system to improve solubility, permeability, stability, and controlled release.

## 1. Introduction

Since their introduction in the 1940s, antibiotics have become a central pillar of healthcare [1]. Antibiotics have been used to prevent people with weakened immune systems succumbing to serious infections; e.g., those occurring during surgical procedures [2]. During the period from 1940 to 1962, more than 100 antibiotics were identified, mainly from microorganisms found in Nature, and thus, this period is called the “Golden Age” of antibiotics [3]. However, the emergence of microorganisms resistant to existing antibiotics has required the continuous development of new antibiotics. From the the 1960s to the 2000s, essentially no new antibiotics were found, and this period is called the “Innovation Gap” [4,5]. During the Innovation Gap, researchers tried to develop and launch various antibiotics by modifying existing compounds. However, concern that antibiotics were being abandoned or discarded due to rapidly acquired tolerance problems before recovery of their development costs led to the contraction of antibiotic research. For example, *Staphylococcus aureus* (*S. aureus*) could be treated with first-generation penicillin, but penicillin-resistant *S. aureus* appeared within a year. Subsequently, new methicillin-based antibiotics were developed to treat penicillin-resistant *S. aureus*, but methicillin-resistant *S. aureus* (MRSA) emerged in 1986. The newly developed vancomycin played a major role in the treatment of MRSA. However, the use of vancomycin caused the emergence of vancomycin-resistant *S. aureus* (VRSA) [5]. Therefore, the use of newly developed antibiotics can lead to the rapid emergence of new antibiotic-resistant strains. This also means that with greater antibiotic overuse, the greater the likelihood that antibiotic-resistant bacteria will become prevalent [6]. Two factors that cause an increase in antibiotic consumption worldwide are increased income and increased demand for animal protein. Increased income has the effect of prolonging the life of individuals, but it also promotes bacterial drug resistance through the increased use of antibiotics. An increase in demand for animal protein leads to an increase in livestock production, which further increases the use of antibiotics in the agricultural and livestock industries, and ultimately also leads to antibiotic resistance [7]. However, as the use of antibiotics continues to expand in each country, the antibiotic market itself is steadily growing. Since the 2000s, technology and industry have developed, and new antibiotics have been developed. However, the success rate of new antibiotics is still low, at 11.8%, and the development period for antibiotics is at least 11 years [8,9]. Thus, if a carrier capable of promoting the effect of existing antibiotics is developed or an antibiotic effect is improved by combining existing antibiotics, the development period can be shortened and the development success rate can increase.

In general, antibiotic resistance refers to bacteria becoming resistant to antibiotics that have previously exhibited an effective antibacterial action [10]. Through natural mutation of a bacterial chromosomal gene or exogenous gene such as a plasmid, the bacterium can acquire resistance to an antibiotic [11]. The resistance gene is expressed and provides resistance to antibiotics through mechanisms such as: (1) enzymatic inactivation, (2) modification of the antibiotic target, and (3) efflux pumping [12]. Thus, new antibiotics should be able to neutralize these bacterial defense mechanisms. For example, the stability of antibiotics could be improved by modifying the structure of the antibiotic to enhance its stability against the enzyme that destroys it or by treating it with a substance that inhibits the activity of the degradative enzyme [13,14,15,16]. Another strategy to overcome resistance is to improve delivery or accessibility, so antibiotics can act effectively at the site of action. For example, liposome carriers for hydrophobic antibiotics such as ethambutol, rifampin, capreomycin, and resorcinomycin A have been reported [17,18].

Carbohydrates are the most abundant biomolecules in Nature, mainly found as polysaccharides that play crucial roles in both animal and vegetable life. They are also molecular building blocks of cells [19], and capable of molecular recognition [20]. Most carbohydrates found in Nature are generally recognized as safe (GRAS) substances [21] and can be used in various applications including foods, cosmetics, and pharmaceuticals [22]. In particular, several types of carbohydrate form complexes with other substances via host-guest complexation, and can change physicochemical properties of these substances, such as enhancing their solubility, stability, permeability, and bioavailability [23]. In host-guest chemistry, host molecules can complex with guest molecules through non-covalent hydrogen bonds, van der Waals forces, hydrophobic interactions, and electrovalent bonds [24]. Cyclodextrins (CDs) are cyclic α-1,4 glucans produced from starch using cyclodextrin glucosyl transferase (CGTase), which is produced by a large number of microorganisms [25]. The most common CDs are α CD, β CD, and γ CD, consisting of 6-, 7-, and 8-glucose units, respectively. They have a hydrophilic surface and a hydrophobic cavity that can be complexed with hydrophobic molecules (guests) via host-guest complexation [26]. CDs are the best-known carbohydrate-based host molecules used in food, cosmetic, sensor, biomedical, and pharmaceutical applications [27]. Among them, β CD is the most used in research, but its water solubility is the lowest of all CDs because of its intramolecular hydrogen-bonding patterns [25]. Chemical modification of CDs also improves their complexation ability against specific substances. Thus, β CD and its derivatives can be used for hydrophobic drug delivery systems to enhance drug solubility and bioavailability [28].

Cyclosophoraoses (Cys), with linkage and polymerization patterns that differ from those of CDs, also have host-guest complexation abilities [29]. Cys are cyclic β-(1,2)-glucans containing 10–40 glucose units and, which are produced from *Rhizobium* and *Agrobacterium* spp. [30]. Cys have higher water solubility (1350 mg/mL at 25 °C) than β CD (16 mg/mL at 25 °C) due to their flexible β-(1,2)-glycosidic linkage and large ring structure [31]. Therefore, although there are few reports, Cys can also be used for hydrophobic drug delivery systems [23].

Linear oligosaccharides can provide amphiphilicity because of their hydrophilic and hydrophobic surfaces on sugar backbones, and solubilize lipophilic compounds in water [32,33,34]. *Sinorhizobium meliloti* produces low-molecular-weight (LMW) succinoglycans (SCs) which are monomers, dimers, and trimers of the octasaccharide repeating unit [35]. The main chain is composed of β-1,3-, β-1,4-, and β-1,6-linked octasaccharide units, containing one galactose at the reducing end and seven glucose units. They also contain pyruvyl, acetyl, and succinyl substituents on the sugar backbones. Based on their hydrophobicity, succinoglycans can exhibit hydrophobic interactions with fluorescent probes via an induced-fit type adjustment [36], and this has been applied to antibacterial drugs. Furthermore, linear β-glucan from *Pseudomonas syringae* (*P. syringae*) has been reported to form complexes with aminoglycoside antibiotics. The linear β-glucans are β-1,2- and β-1,6-branched glucans containing 6–12 glucose residues [37].

In a previous study, β CD/antibiotic complexes had lower MIC values than antibiotics alone for some bacterial strains, since β CD or β CD derivatives can improve the stability and permeability of antibiotics [38]. However, the MIC values of these complexes remained similar to those of antibiotics alone. Furthermore, there have been few reports of enhancing the antibacterial activity of antibiotics via host-guest complexation. This paper reviews recent studies describing the enhanced antibacterial activity of antibiotics using carbohydrate-based host molecules including CDs, Cys, linear oligosaccharides, and their derivatives.

## 2. Mechanisms of Host-Guest Complexation in Drug Delivery

Host molecules can enhance the apparent water solubility of guest molecules by complexation. This complexation is controlled by a stability constant, *K_c_* [24]. Most drugs form 1:1 complexes with host molecules [39] and the stability constant (*K*_1:1_) of 1:1 complex can be defined by following equation:

$$K_{c}=K_{1:1}=\frac{{\left[Guest\right]}_{complex}}{{\left[Host\right]}_{free}{\left[Guest\right]}_{free}}$$

where [*Guest*]*_complex_* represents the concentration of guest molecules in the complex form, and [*Guest*]*_free_* and [*Host*]*_free_* represent the free guest and free host molecule concentration, respectively. The *K*_1:1_ can be obtained through phase solubility analysis. If the drug forms a 1:1 complex with host molecules, the drug solubility increases linearly as the concentration of host molecule increases. In this case, the slopes of the linear curve is always less than unity [40], and *K*_1:1_ can be calculated by following equation:

$$K_{1:1}\left(M^{-1}\right)=\frac{slope}{{\left[S\right]}_{0}\left(1-slope\right)}$$

The slope is obtained by phase solubility analysis, and [*S*]_0_ represents the intrinsic solubility of the drug in water. Most values of *K*_1:1_ fall within the range 1000–20,000 [39], high values of *K*_1:1_ imply a strong interaction between drug and host molecules. Despite this strong interaction, the kinetics of formation and dissociation of complexes between host and drug molecules is fast. The half-life of complex formation and dissociation is less than one second, and the velocity approaches that of the diffusion phenomenon [41,42]. The release of drug from complexes is expected to be rapid, and occurs by simple dissociation, competitive displacement, tissue uptake, and protein binding. Moreover, once a weakly bound drug is diluted, it can quickly dissociate from the host molecule [43,44].

Carbohydrate-based host molecules are relatively large (molecular weights ranging from 1000 to 5000). Host molecules hardly permeate biological membranes under normal conditions. In general, host molecules act as drug carriers by keeping hydrophobic drug molecules in solution and delivering them to the surface of biological membranes [45]. Unlike conventional penetration enhancers, such as alcohol and fatty acids, host molecules increase drug availability on membrane surfaces without disrupting the lipid layers of the biological barriers.

The complexation of host and guest molecules is affected by the structure and conformation of each molecule [46]. Most studies have focused on these properties of host molecules such as CDs. Natural α, β, and γ CD consist of six, seven, and eight glucose units, respectively. The cavity diameters of α, β, and γ CD are ~5.3, ~6.5, and ~8.3 Å, respectively [47]. The cavity diameter of CDs determines critical factors in host-guest complexation. Combinations of several other parameters, including hydrophobicity, chirality, size, and shape of guest molecules, also influence complex formation between hosts and guests [48]. Thus, modification of host molecules can improve their complexation ability with specific drugs. These modified host molecules are classified into hydrophilic, hydrophobic, and ionic derivatives. Hydrophilic derivatives, such as those having undergone hydroxypropyl and glucosyl modification, have been administered intravenously to rats, and were found to disperse rapidly from plasma [49]. Alkylated and acrylated hydrophobic derivatives will be useful as sustained-release carriers for water-soluble drugs [50,51]. In anionic derivatives, cationic drugs can be effectively complexed by electrostatic effects [52,53].

Titration methods, using UV/Vis, fluorescence, NMR spectroscopy, and isothermal titration calorimetry (ITC), can be performed to reliably quantify host-guest complexation [54,55,56]. From these analyses, we can obtain the entropy or enthalpy of host-guest complexation. Further information about the structure and conformation of host-guest complexes can also be obtained through 2D NMR analysis. Molecular modeling can be used to define the conformation of complexes and to design compatible host molecules [57]. Computational molecular modeling studies correlate well with experimental results, and provide configurational entropy and energy components of complex formation [46]. Although molecular modeling of host and drug molecules has been studied, there have been very few reports using computer molecular modeling to design hosts that can complex with specific drugs. It is reasonable because the crystallographic data of carbohydrate-based host molecules are not sufficiently accumulated with the exception of the disaccharides and CDs. If the dynamic three-dimensional structure can be established using NMR spectroscopy, solution scattering, cryo-EM, and X-ray crystallography [58], the design of host molecules for the target drug will be facilitated.

## 3. Mechanisms of Antibacterial Resistance

Antibiotics are classified and named mainly according to their chemical structure and functional groups [59]. The β-lactam antibiotics have a basic structure containing a β-lactam ring and bind to penicillin-binding protein (PBP). They act on the bacterial cell wall and inhibit cell wall synthesis. Some bacteria possessing β-lactamase can break down the β-lactam ring and show antibiotic tolerance or resistance by inducing deformation of PBP [60]. Glycopeptide antibiotics have macromolecules composed of sugar and amino acid. They include vancomycin and teicoplanin, and bind to murine monomers that are constituents of the bacterial cell wall (peptidoglycan) and inhibit cell wall synthesis [61]. Glycopeptide-resistant *Enterococci* possess a modified peptidoglycan terminus, which decreases the affinity of vancomycin for the target peptidoglycan [62]. Vancomycin-tolerant *Staphylococci* have a thickened cell wall, compared with vancomycin-sensitive *Staphylococci* [63]. The tetracycline antibiotics have four hexagonal rings (tetra + cycline). They are protein synthesis inhibitors with excellent antimicrobial efficacy against various kinds of bacteria. This class of antibiotics binds to ribosomes and inhibits protein synthesis. However, the tetracycline antibiotics are easily degraded, toxic, and induce the emergence of resistant bacteria by ribosomal mutation [64,65]. In addition, new quinolone and rifamycin antibiotics inhibit bacterial DNA and RNA synthesis. Most antibiotic-resistant bacteria will acquire resistance by mutating the antibiotic target site. Another important resistance mechanism is efflux of the antibiotics through the cell membrane, which reduces the intracellular concentration of the antibiotic and prevents it from reaching the target site [66].

The misuse of antibiotics promotes the emergence of antibiotic-resistant bacteria. Although multiple antibiotics can be used for the eradication of antibiotic-resistant bacteria, this method encourages the development of multidrug-resistant strains. Therefore, it is necessary to develop a new class of antibiotics or agents that are rapidly effective against antibiotic-resistant bacteria [67]. In this regard, host-guest complex systems are expected to offer several advantages. First, carbohydrate-based host molecules can enhance the solubility of poorly soluble antibiotics via inclusion complexation, and the system can then kill bacteria more effectively and quickly without allowing the time needed to acquire antibiotic resistance. Second, host-guest systems can protect antibiotics from antibiotic-degrading enzymes. Third, carbohydrate-based host molecules may change the conformation of antibiotics and increase their affinity for the modified active site on antibiotic-resistant bacteria. Fourth, by changing the permeability of antibiotics, it can neutralize the efflux pump action or mutated membrane of antibiotic-resistant- bacteria. For all of these reasons, carbohydrate-based host molecules may offer advantages when treating antibiotic-resistant bacteria.

## 4. β-Cyclodextrin and Its Derivatives

### 4.1. Methicillin/per-6(4-methoxylbenzyl)-amino-6-deoxy-β-cyclodextrin Complex

Methicillin was first developed in 1959 as a β-lactam antibiotic. However, methicillin-resistant *S. aureus* were found in England two years later and methicillin was no longer prescribed to patients [68]. Methicillin is insensitive to the enzyme β-lactamase (penicillinase) that neutralizes the activity of penicillin. Thus, methicillin could be used to treat penicillin-resistant bacteria, which release penicillinase [69]. Generally, the effect of β-lactam antibiotics on bacteria depends on their ability to reach and bind penicillin-binding proteins (PBP). β-lactam antibiotics bind to PBP to block the final transpeptidation of the initial peptidoglycan layer and interfere with cell wall synthesis [70]. However, MRSA strains generate other PBPs, such as PBP2a and PBPA, which represent a structural change in the active site [71]. The affinity of the β-lactam antibiotics for PBPs whose structure has been changed is reduced, and cell wall synthesis is not inhibited.

**Figure 1:**
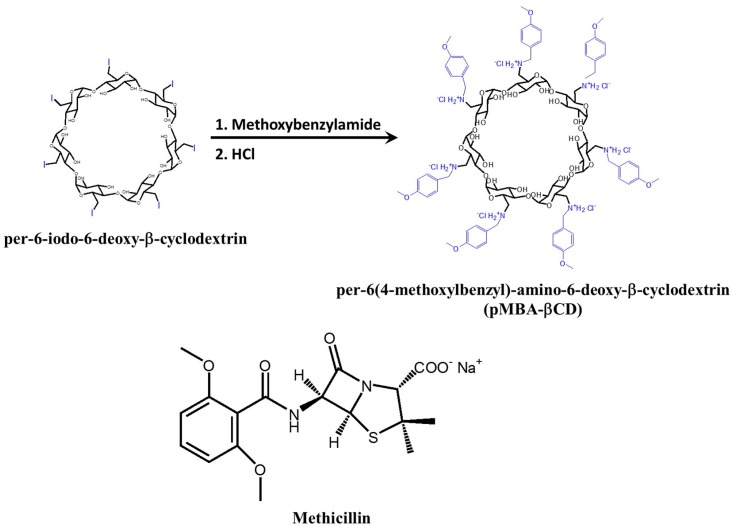
Synthesis of pMBA-βCD starting from per-6-iodo-6 β-cyclodextrin and the chemical structure of methicillin.

Deng et al. designed per-6(4-methoxylbenzyl)-amino-6-deoxy-β-cyclodextrin (pMBA-βCD) for complexation with methicillin [72]. The pMBA-βCD was synthesized from per-6-iodo-6-deoxy-β-cyclodextrin prepared from β CD (Figure 1). The complex formed between pMBA-βCD and methicillin was investigated through ^1^H-NMR and NOESY experiments. pMBA-βCD formed a 1:1 complex with methicillin, and the β-lactam residue of methicillin was encapsulated into a cavity. The negative charge of the β-lactam residue was reduced by the positive nitrogen ion on pMBA-βCD, which can increase the complexation ability and water solubility of methicillin. The MIC values of pMBA-βCD/methicillin and hydroxypropyl-βCD (HP-βCD)/methicillin complexes were evaluated against two MRSA strains, MRSA COL and MRSA USA300. The MIC values of pMBA-βCD/methicillin was decreased 30–65-fold compared with that of methicillin alone (Table 1). The specific conformation of the pMBA-βCD/methicillin complex could provide better affinity for active β-lactam residues to accommodate the modified PBP2a.

### 4.2. Ciprofloxacin/mono-6-Deoxy-6-aminoethylamino-β-cyclodextrin Complex

Ciprofloxacin is a fluoroquinolone antibiotic that has a broad-spectrum bactericidal effect by interfering with bacterial DNA replication [71]. Since the activity of ciprofloxacin depends on physico-chemical properties, such as hydrophobicity and degree of ionization, previous studies have attempted to increase its solubility for effective drug delivery. The salt form of ciprofloxacin provides higher solubility in water, but its solubility in buffer is lowered [73]. Although β CD and HP-βCD have also been used to increase the solubility of ciprofloxacin via solid inclusion complexation [74,75], the antibacterial activity of CD/ciprofloxacin complex forms has not been evaluated.

**Figure 2:**
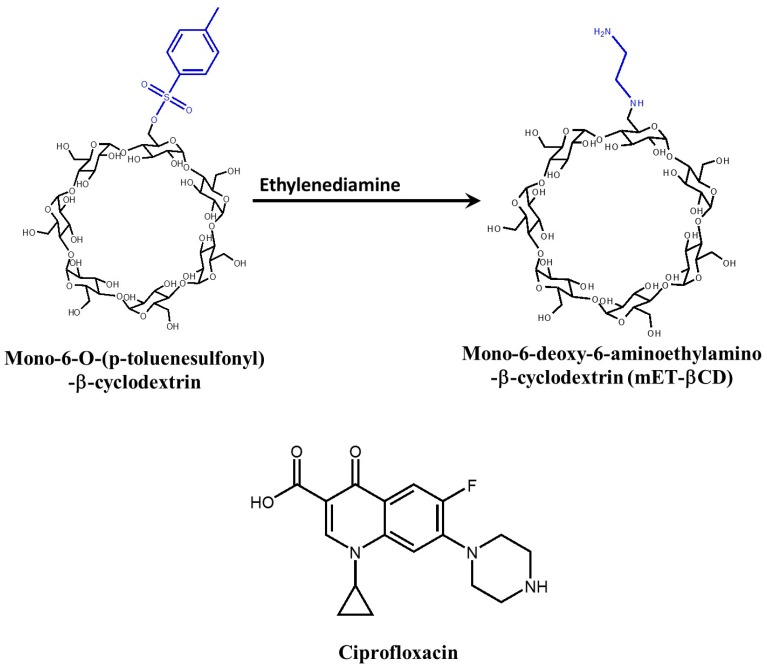
Synthesis of mET-βCD starting from mono-6-*O*-(*p*-toluenesulfonyl)-β-cyclodextrin and the chemical structure of ciprofloxacin.

Mono-6-deoxy-6-aminoethylamino-β-cyclodextrin (mET-βCD) can enhance the solubility and antibacterial activity of ciprofloxacin (Figure 2) [76]. Interestingly, the cavity of mET-βCD is more oval-shaped, compared with native β CD, as determined by NOESY NMR analysis and molecular modeling. From NOESY NMR analysis of mET-βCD, the ethylenediamine moiety of mET-βCD induces stable hydrogen bonding with primary hydroxyls of β CD, leading to the distortion of the circular cavity of β CD. The formation of this oval-shaped cavity causes quinolone and the cyclopropyl groups of ciprofloxacin to be embedded in the mET-βCD cavity. The stability constant of ciprofloxacin with mET-βCD was 21-fold higher than that of ciprofloxacin with β CD (Table 2). Furthermore, growth inhibition of MRSA by ciprofloxacin/mET-βCD significantly increased. These results indicate that mET-βCD effectively increased the solubility and bioavailability of ciprofloxacin.

### 4.3. Butylparaben and Triclosan with Cationic β-Cyclodextrin Polymer

Butylparaben and triclosan can inhibit the growth of a broad range of microorganisms. Butylparaben inhibits cellular ATPase and phosphotransferase [79] and they mainly used as preservative in various area. Triclosan can block the synthesis of lipids [80] and inhibit the NADPH-dependent enoyl-acyl carrier protein reductase in bacteria [81]. Both antibiotics have been widely used in the cosmetic, drug, and food industries. However, they are non-ionic compounds and have low solubility in water, which limits their application.

**Figure 3:**
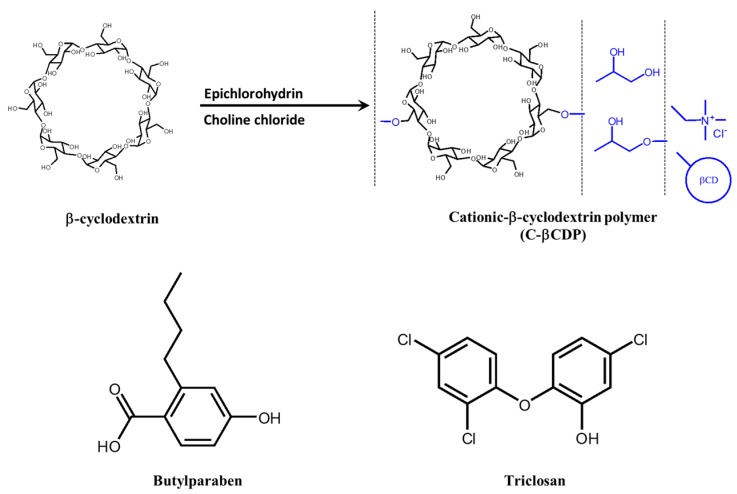
Synthesis of cationic β-cyclodextrin polymer and chemical structure of butylparaben and triclosan.

Cyclodextrin polymers have high solubility in water and the capability to complex with hydrophobic compounds of relatively large molecular size. This is due to the cooperation of two adjacent CD structures on the polymer chain [82]. In particular, cationic β-cyclodextrin polymer (C-βCDP) has high molecular weight and low cationic charge density, exhibiting good drug complexation and dissolution abilities [83]. C-βCDP is synthesized with epichlorohydrin and choline chloride, and utilized to overcome the poor aqueous solubility of butylparaben and triclosan (Figure 3) [84]. In phase-solubility studies of C-βCDP with butylparaben and triclosan, the water solubility of butylparaben and triclosan significantly increased as a function of the C-βCDP concentration. The MIC value of triclosan/C-βCDP complex against *E. coli* ATCC 11299 slightly decreased compared with previous reports [85,86]. However, the MIC value of the butylparaben/C-βCDP complex was dramatically lower than in other reports [87,88]. Since C-βCDP has cationic properties, it can effectively approach the negatively charged surface of bacterial membranes via electrostatic attraction. However, it is not clear whether the antibiotic/C-βCDP complex can act on antibiotic-resistant bacteria, because *E. coli* ATCC 11299 is not an antibiotic-resistant bacterium. Nevertheless, this result suggests that delivery of non-ionic antibiotics can be improved by forming complexes with C-βCDP.

### 4.4. Sugar-Grafted β-Cyclodextrin for Delivering Antibiotics

**Figure 4:**
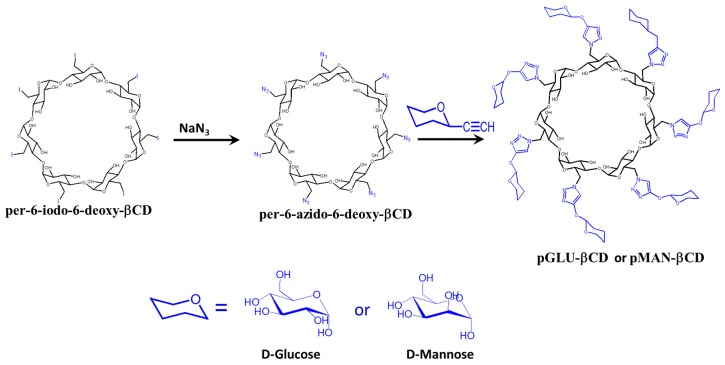
Synthesis of compound cationic sugar-grafted β CD.

Sugar-grafted β CD was designed as an antibiotic carrier [89]. Li et al. prepared d-mannose (pMAN-βCD) and d-glucose-grafted β-cyclodextrin (pGLU-βCD) via a click reaction (Figure 4). Glucose and mannose are carbon sources for bacteria, and can easily penetrate into bacterial cells through cell membrane sugar transporters [90].

Thus, sugar-grafted β CD can be used for effective delivery of antibiotic to cells by using the attached sugar as a chemo-attractant for the bacteria. The antibacterial activities of pMAN-βCD and pGLU-βCD with erythromycin, rifampicin, and ciprofloxacin were investigated against *S. aureus* 25293, *Escheriachia coli*, and *Pseudomonas aeruginosa*.

The MIC values of sugar-grafted β CD/antibiotic complexes against the tested bacteria were significantly lower than those of antibiotics alone. The pMAN-βCD/antibiotic complex was able to inhibit antibiotic-resistant bacteria. The MIC value of erythromycin alone for efflux-proficient *P. aeruginosa* was 8 times higher than for *P. aeruginosa* without the efflux pump, indicating that the efflux pump resulted in a significant increase in erythromycin resistance in *P. aeruginosa*. However, the MIC value of the pMAN-βCD/erythromycin complex for efflux-proficient *P. aeruginosa* was 3.3 times lower compared with erythromycin alone. In contrast, the corresponding MIC value reduction for *P. aeruginosa* without the efflux pump was only 1.7 times lower. These results indicate that the pMAN-βCD/erythromycin complex has potent antibacterial activity against efflux-proficient bacteria. The antibiotic delivery mechanism of pMAN-βCD is not clear, but it is possible that pMAN-βCD delivers the antibiotic effectively into the bacterial cytoplasm, leading to increased intracellular drug concentrations. The sugar moiety of β CD also plays an important role in antibiotic delivery.

Furthermore, the growth of *E. coli* was inhibited for 3 days in erythromycin-treated culture media, but pMAN-βCD/erythromycin complex-treated culture media suppressed *E. coli* growth even after 18 days. These results were also recorded in other microbial growth experiments, indicating that pMAN-βCD enhances the stability of erythromycin and preserves its antibacterial activity for a long period.

## 5. Cyclosophoroase and Its Derivatives

Although the use of Cys for improving the antibacterial activity of antibiotics has not been reported, Cys can improve the solubility and bioavailability of flavonoids [23,91]. Flavonoids are polyphenols, commonly found in natural products such as fruit, vegetables, seeds, flowers, propolis, and honey [92]. They possess antifungal, antiviral, anti-inflammatory, anti-oxidant, anti-cancer, and antibacterial activities.

**Figure 5:**
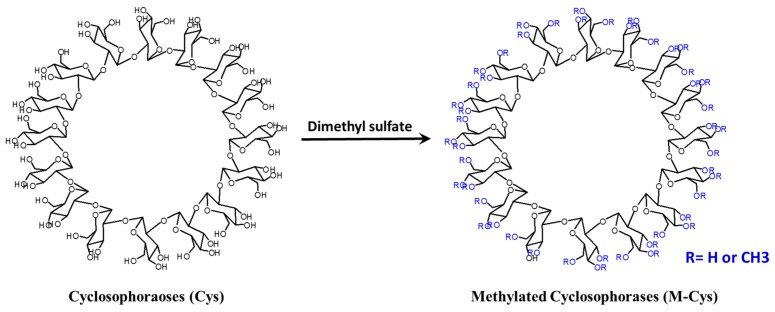
Synthesis of methylated Cys starting from Cys.

Recently, various flavonoids have been used against antibiotic-resistant bacteria [93,94,95,96]. In this setting, improving the solubility and bioavailability of flavonoids can contribute to the elimination of antibiotic-resistant bacteria. Since they have limitations in terms of low solubility in water and low bioavailability [97,98], we have improved the solubility and bioavailability of flavonoids with antimicrobial activity using Cys or Cys-derivative complexes (Figure 5).

Galangin is known to inhibit the L1 metallo-β-lactamase of *Stenotrophomonas maltophilia* and MRSA [94,99]. However, the water solubility of galangin is extremely low (~14.3 μg/mL), thus limiting its application. Kim et al. reported enhanced solubility of galangin using methylated cyclosophoraose (M-Cys) [100]. The galangin/M-Cys complex was suitable for 1:1 complexation, as determined by the continuous variation plot method. The stability constants of galangin with β CD, dimethyl β CD, Cys, and M-Cys were 988, 2690, 3289 and 5534 M^−1^, respectively. The solubility of galangin was enhanced 5.6-fold using 1 mM of M-Cys. Furthermore, M-Cys enhanced the anti-cancer activity of galangin against human cervical carcinoma cells. Given this result, we expect pharmacological activities to be improved by M-Cys complexation. In succession, a Cys/cellulose hydrogel using epichlorohydrin was developed as a delivery system for galangin [31]. Galangin-loaded Cys/cellulose hydrogel showed much longer antibacterial activity than CD/cellulose and cellulose hydrogels. This result also indicates that the antibacterial activity of galangin in Cys complexes was preserved for a long period.

Naringenin, responsible for the bitter taste of grapefruit, inhibits the cytoplasmic membrane function of microbes at high concentrations [93]. It also has poor solubility in water and minimal bioavailability due to its largely hydrophobic ring structure. The solubility of naringenin was enhanced approximately 7.1-fold using 10 mM of Cys [101]. From ^1^H-NMR analysis, the chemical shift changes at the H-6 and H-8 protons on the A-ring of naringenin occurred by complexation with Cys. This result suggested that Cys can regioselectively interact with naringenin.

Luteolin can be used in combination with β-lactam antibiotics for treating MRSA infection [102]. Luteolin can affect the membrane permeability of *S. aureus*, but does not disrupt the membrane directly [103]. The solubility of luteolin can be improved by forming complexes with Cys [104]. The stability constants of Cys and β CD are 332,933 and 2383 M^−1^, respectively, indicating a 139-fold enhancement by complexation with Cys, comparing with β CD.

## 6. Linear Oligosaccharides

**Figure 6:**
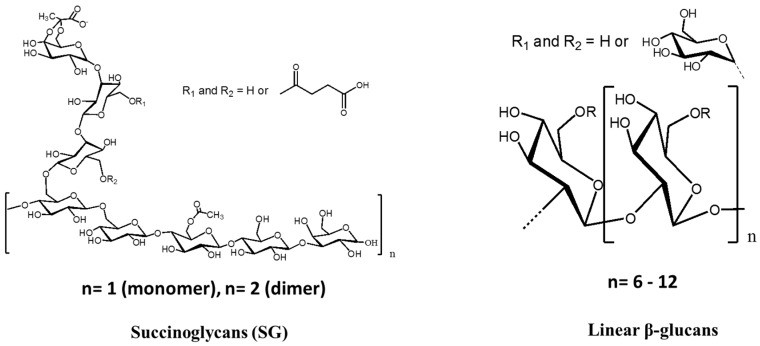
Structure of succinoglycan (SG) and liner β-glucans.

Polysaccharides are generally natural polymers such as chitosan, alginate, cellulose, and agar and are used in established drug delivery systems [105,106]. In recent studies, linear oligosaccharides have also been used in drug delivery systems for host-guest complexation [23,33]. In this section, we summarize the study of linear oligosaccharide complexes with antibiotics and flavonoids (Figure 6).

Succinoglycan (SG) dimers can complex with pyrimethamine to form an antibacterial drug [33]. Pyrimethamine inhibits dihydrofolate reductase (DHFR), which is an enzyme essential for the conversion of folic acid into folinic acid during nucleic acid biosynthesis in protozoa. However, a large amount is required to sufficiently suppress the growth of parasites since the water solubility of the pyrimethamine is quite low. Such overdose of antibiotics can lead to the emergence of new antibiotic-resistant strains. Kim et al. purified the SG monomers 1, 2 and 3 and dimers 1, 2, 3 and 4 and evaluated the enhancement of pyrimethamine solubility resulting from its complexation with SG. SG dimer 3 exhibited the greatest effect, with only 1.2 mM increasing the solubility of pyrimethamine 42-fold after complex formation. The stability constants were ranked in the following order: SC dimer 3 > SG dimer 4 > SG dimer 2 > HP-βCD > SG monomer 3 > α CD > SG dimer 1 > SG monomer 1 > SG monomer 2. The SG dimer 3/pyrimethamine complex had a 1:2 stoichiometric conformation. In molecular docking simulations, SG dimer 3 contained two binding sites for pyrimethamine. The chlorophenyl residue of pyrimethamine and the sugar rings of SC dimer 3 were in contact. In the case of the pyrimethamine ethyl group, the SG dimer 3 succinyl group was located nearby. Although the antibacterial activity was not evaluated, SG dimer 3 greatly enhanced the water solubility. Considering this finding, the pyrimethamine/SG dimer 3 complex may have potential for clinical applications.

Tobramycin is an aminoglycoside antibiotic and has pharmacokinetic properties similar to those of gentamicin, but the antibacterial activity of tobramycin was more active than gentamicin for *P. aeruginosa* and gentamicin-resistant strains [107]. Tobramycin prevents ribosomal complex formation in microbes, by binding the bacterial ribosomes. Interestingly, the molecular interaction between tobramycin and linear β-glucan produced from *P. syringae* can be demonstrated using NMR spectroscopy [37]. Tobramycin/linear β-glucan complex has been determined to have a 1:1 conformation. Considering that linear β-glucan exists in the periplasmic space of *P. syringae*, antibiotic resistance due to the periplasmic glucans might be expected. On the one hand, other linear β-glucans have the potential to enhance the antibacterial activity and solubility of antibiotics.

## 7. Conclusions

The emergence of antibiotic-resistant bacterial strains is a serious problem worldwide. The major cause of this emergence is the misuse and overdosing of antibiotics, practices that are monitored and regulated in many countries [8]. Various new antibiotics are being developed using modern technology. However, new antibiotics always present the opportunity for the emergence of new antibiotic-resistant bacteria. Therefore, it is necessary to find a way to minimize the use of antibiotics and maximize their effectiveness. Carbohydrate-based host molecules are able to enhance the water solubility and antibacterial activity of antibiotics. Specific host molecules also improve the stability of antibiotics in the face of challenging environmental conditions and cleavage enzymes. In this respect, research on carbohydrate-based host-guest complexes is another strategy to reduce the misuse of antibiotics. In addition, since recently developed antibiotics contain two or more entities to benefit from their synergetic effects, host molecules other than CD that maximize the effectiveness of each antibiotic are needed. Currently, a product that uses carbohydrate-based host molecules as a drug delivery system are on the market. Cefotiam hexetil HCl (Pansporin T, Takeda Pharmaceutical Company Ltd., Osaka, Japan), itraconazole (Sporanox^®^, Janssen Pharmaceuticals, Beerse, Belgium), chloramphenicol (Clorocil^®^, EDOL, Linda-a-Velha, Portugal), and voriconazole (Vfend^®^, Pfizer Inc., New York, NY, USA) are commercial CD-antimicrobial medicines. Since these products increase the bioavailability of the drugs using several CD derivatives, the development of an effective antibiotic delivery system using carbohydrate-based host molecules might have potential in terms of antibiotic therapy.

## Figures and Tables

**Table 1:** Antibacterial activities for methicillin and pMBA-βCD alone and in complex form (MIC, mg/L).

Test Sample	MRSA COL	MRSA USA300
Methicillin	>128	>128
HP-βCD/methicillin	>64	>64
pMBA-βCD/methicillin	2.0–4.0	2.0–4.0

**Table 2:** Stability constants of ciprofloxacin with β CD derivatives and antibacterial activity of ciprofloxacin/β CD derivative complexes against MRSA.

Test Sample	Stability Constant (M^−1^)	Antibacterial Activity ^1^
Ciprofloxacin	-	5.78 [76]
Β CD	29.84 [76]	5.58 [76]
	29.1 [77]	
HP-βCD	278 [75]	
mET-βCD	627.3 [76]	8.825 [76]

^1^ Antibacterial activity was calculated by following equation; antibacterial activity = −log (*N*/*N*_0_), where, *N* was the CFU/mL of bacterial suspension on the sample and *N*_0_ was the initial CFU/mL [78].
